# Evaluation of Thyroid Hormone Status: Are Simultaneous TSH and FT4 Tests Necessary? Analysis of Thyroid Function Test Results Taken From the Greater Manchester Care Record 2010–2023

**DOI:** 10.1111/cen.70012

**Published:** 2025-08-19

**Authors:** Michael Stedman, Peter Taylor, Ian Halsall, David Halsall, Buchi Okosieme, Suhani Bahl, Lakdasa Premawardhana, Colin Dayan, Anthony A. Fryer, Adrian Heald

**Affiliations:** ^1^ Res Consortium Andover UK; ^2^ Cardiff University School of Medicine Cardiff UK; ^3^ Addenbrooke's Hospital Cambridge UK; ^4^ Keele University Keele UK; ^5^ Salford Royal Hospital Salford UK; ^6^ University of Manchester Manchester UK

To the Editor,

There continues to be much discussion around optimization of thyroid hormone status in hypothyroid individuals. The ideal therapeutic goal in hypothyroidism would be to restore clinical and biochemical euthyroidism via physiologic thyroid hormone replacement. The relation between circulating thyroid hormone levels (FreeT4 [fT4] and FreeT3) and thyroid stimulating hormone (TSH) is integral to this. This concept may seem straightforward, but there are subtleties that have only recently been recognized [1].

Over 10 million thyroid function tests (TFT) are undertaken in England annually. To reduce test numbers, laboratories very often measure TSH and then only reflex measure fT4 if TSH results fall outside the reference range (RR) [2].

We analysed the relation between fT4 and TSH results for patients both on and off thyroid medication using a city‐wide population database.

Simultaneous TSH and fT4 results from 47,869 diagnosed hypothyroid individuals (including information on dose) and 393,101 untreated/euthyroid individuals who had been tested once or twice were included. Data were extracted from the Greater Manchester Care Record (GMCR) [3].

The data used in the analyses presented was obtained with the permission of the Greater Manchester Care Record Board (reference number R 2023 065) and was fully anonymised before being made available to the investigators.

Diagnostic data were validated and cleaned before analysis. Only patients with a coded diagnosis of primary hypothyroidism were included.

Our findings showed:

***Untreated individuals*** (Table 1)

**Table 1:** TSH versus fT4 apportioned by TSH category.

TSH versus FfT4 apportioned by TSH category		fT4 < 9	fT4 9–25	fT4 > 25	Total
UNTREATED	TSH < 0.4	48	13,146	115	13,309
	TSH 0.4–4	**3319**	403,206	**175**	406,700
	TSH > 4	625	31,819	10	32,454
	Total	3992	448,171	300	452,463
TREATED	TSH < 0.4	201	92,574	10,195	102,970
	TSH 0.4–4	**1368**	172,400	**3065**	176,833
	TSH > 4	7499	118,910	863	127,272
	Total	9068	383,884	14,123	407,075

*Note:* Bold values are statistically significant.

Of the 452,463 results in the untreated/euthyroid population (*n* = 393,101 people), 89.9% (406,700) were within the TSH‐RR and 99.1% (448,171) were within the fT4‐RR.

Of those 406,700 with a TSH within the RR, 3319 (0.82%) had a fT4 < 9 and 175 (0.043%) had a fT4 > 25. These account for 83.1% of all results with a fT4 < 9 and 58.3% of all those with a fT4 > 25.

**Both these groups might not receive a reflex fT4 result if REFLEX testing were applied.**

***Treated individuals*** (Table 1)

Of 407,075 results in the treated population (*n* = 47,869 people), 176,833 (43.4%) were in TSH‐RR.

Of those 176,833 with a TSH within the RR, 1368 (0.77%) had a fT4 < 9 and 3065 (1.73%) had a fT4 > 25. These account for 15.1% of all results with a fT4 < 9 and 21.7% of all those with a fT4 > 25.

**Both these groups might not receive a reflex fT4 result if REFLEX testing was applied.**

These effects were more prevalent in people taking a higher dose of levothyroxine.

When only those with normal TSH results were considered, we found that in the untreated cohort, 0.043% had an FT4 > 25 pmol/L. However, within the population being treated with levothyroxine, this was 1.73%, showing that the relative risk of high FT4 in treated individuals was *40 times* higher than in the untreated.

In conclusion, there are marked differences in the TSH–fT4 relationship between treated and untreated individuals, accentuated as levothyroxine dose increases. In levothyroxine treated individuals, we found a large number of people with a normal TSH but elevated fT4. This follows on from a previous analysis of thyroid function testing where we reported that the majority of TFT requests were requested outside recommended intervals and within‐practice variability was high [4].

We acknowledge previous work suggesting that combined fT4 and TSH testing is not necessary, with the alternative being a two‐step approach based on first testing TSH [5]. However, in this representative large community sample, we have identified a large number of people treated with levothyroxine who have a fT4 outside the laboratory RR.

There is variability in relation to the way that thyroid hormone status assessment is decided. However, the laboratory can lead on best practice. We here have provided evidence that thyroid function testing for individuals on levothyroxine replacement should include a check of fT4 as well as TSH. Such an approach, if adopted, can be enabled by the simple strategy of requiring the clinician requesting the test to state whether or not the patient is taking thyroid hormone replacement with a mandatory tick box.

Regarding cost estimates, while assay costs will be subject to some variation between laboratories, our estimate is for an additional £0.80 (Euro 0.93) of fT4 estimation to be added to TSH (£1.00) (Euro 1.16) for the cost of TSH alone, giving a total cost of £1.80 (Euro 2.09) for fT4 and TSH to be assayed together.

We accept that we do not have sufficient data to support a universal recommendation. Given the possible risks associated with prolonged exposure to high and low fT4 levels [6, 7], we suggest that where indicated, fT4 and TSH should both be measured, in particular for those treated with levothyroxine, to enable thyroid hormone replacement dose adjustment as appropriate. In any case, if pituitary/hypothalamic disease is suspected fT4 and TSH should be checked in order not to miss pituitary insufficiency [8].
